# Coverage of malaria protection in pregnant women in sub-Saharan Africa: a synthesis and analysis of national survey data

**DOI:** 10.1016/S1473-3099(10)70295-4

**Published:** 2011-03

**Authors:** Anna Maria van Eijk, Jenny Hill, Victor A Alegana, Viola Kirui, Peter W Gething, Feiko O ter Kuile, Robert W Snow

**Affiliations:** a Child and Reproductive Health Group, Liverpool School of Tropical Medicine, Liverpool, UK; b Kenya Medical Research Institute–University of Oxford-Wellcome Trust Collaborative Programme, Kenyatta National Hospital Grounds, Nairobi, Kenya; c Spatial Ecology and Epidemiology Group, Department of Zoology, University of Oxford, Oxford; d Department of Infectious Diseases, Tropical Medicine, and AIDS, Academic Medical Centre, University of Amsterdam, Amsterdam, Netherlands; e Centre for Tropical Medicine, Nuffield Department of Clinical Medicine, Centre for Clinical Vaccinology and Tropical Medicine, University of Oxford, Oxford, UK

## Abstract

**Background:**

Insecticide-treated nets and intermittent preventive treatment with sulfadoxine–pyrimethamine are recommended for the control of malaria during pregnancy in endemic areas in Africa, but there has been no analysis of coverage data at a subnational level. We aimed to synthesise data from national surveys about these interventions, accounting for disparities in malaria risk within national borders.

**Methods:**

We extracted data for specific strategies for malaria control in pregnant women from national malaria policies from endemic countries in Africa. We identified the most recent national household cluster-sample surveys recording intermittent preventive treatment with sulfadoxine–pyrimethamine and use of insecticide-treated nets. We reconciled data to subnational administrative units to construct a model to estimate the number of pregnant women covered by a recommended intervention in 2007.

**Findings:**

45 (96%) of 47 countries surveyed had a policy for distribution of insecticide-treated nets for pregnant women; estimated coverage in 2007 was 4·7 million (17%) of 27·7 million pregnancies at risk of malaria in 32 countries with data. 39 (83%) of 47 countries surveyed had an intermittent preventive treatment policy; in 2007, an estimated 6·4 million (25%) of 25·6 million pregnant women received at least one dose of treatment and 19·8 million (77%) visited an antenatal clinic (31 countries). Estimated coverage was lowest in areas of high-intensity transmission of malaria.

**Interpretation:**

Despite success in a few countries, coverage of insecticide-treated nets and intermittent preventive treatment in pregnant African women is inadequate; increased efforts towards scale-up are needed.

**Funding:**

The Malaria in Pregnancy Consortium and Wellcome Trust.

## Introduction

Malaria infection during pregnancy can lead to very poor outcomes for the mother and child.^1^ In 2007, there were about 32 million pregnancies in malaria-endemic areas in sub-Saharan Africa.^2^ WHO's recommendation for malaria prevention and control during pregnancy in areas of stable malaria transmission in Africa is a package of intermittent preventive treatment and insecticide-treated nets with effective management of clinical malaria and anaemia, which is commonly delivered through collaboration between malaria and reproductive-health programmes.^3^ The recommended drug for intermittent preventive treatment is sulfadoxine–pyrimethamine. These interventions can substantially reduce disease burden and adverse outcomes of malaria in pregnancy,^4^, ^5^, ^6^ and are cheap and cost effective.^7^, ^8^ The Roll Back Malaria initiative aims to ensure that all pregnant women receive intermittent preventive treatment and at least 80% of people at risk from malaria use insecticide-treated nets in areas of high-intensity transmission by 2010, including those who are pregnant.^9^

Achievement of high coverage of intermittent preventive treatment has remained elusive for many countries in sub-Saharan Africa,^10^ despite high use of antenatal care.^11^, ^12^ Specific targeting of pregnant women aimed at increased use of insecticide-treated nets began in sub-Saharan Africa earlier than did intermittent preventive treatment, but coverage is much lower than targets set by the Roll Back Malaria initiative.^10^ Nevertheless, access to antenatal clinics, malaria risk, and population density all vary substantially within most countries and national aggregates of intervention use might mask important targeting of resources to areas with a high malaria risk or inequities in intervention coverage among marginalised communities in such areas. We aimed to analyse subnational intervention coverage congruent with modelled malaria risk to better understand the targeted coverage of insecticide treated nets and intermittent preventive treatment in pregnant women who are most likely to benefit from universal coverage of these interventions.

## Methods

### Data collection and study population

We identified national malaria prevention policies for pregnant women and approximate year of policy adoption from the World Malaria Report^10^ and proposals submitted to the Global Fund to Fight AIDS, Tuberculosis and Malaria.^13^ We obtained data from national policies and contacted national malaria control programmes. We used date of policy adoption to estimate the time between adoption of the policy and national coverage survey year because there were no reliable data for the actual timing of implementation by country. We documented policy ambitions and mechanisms of distribution of insecticide-treated nets within a country, but the available information was insufficient to allow useful analysis. Because of important differences in malaria policy, separate malaria control governance and malaria risk, we report separate data for north Sudan and south Sudan (the semi-autonomous region) and for Tanzania mainland and the islands of Zanzibar.

We obtained data for coverage and use of insecticide-treated nets, intermittent preventive treatment, and antenatal care from national household cluster-sample surveys done as part of demographic and health surveys,^14^ multiple indicator cluster surveys,^15^ malaria indicator surveys,^16^ and a survey done by the Food Security and Nutrition Analysis Unit (FSNAU) in Somalia.^17^ The multistage sampling design from first-level administration (eg, province, state, or region) to national census-defined enumeration clusters is common to all these surveys, and sample sizes are established to provide precision in health and population indicators at the first-level administrative unit (ADMIN1). For three national surveys (Nigeria, Tanzania, and Madagascar), sampling precision was increased to provide estimates of intervention coverage at second-level administrative units (eg, districts) and we have used these sampling units in this analysis. For every country, we identified the most recent surveys that collected information about use of insecticide-treated nets, intermittent preventive treatment, and antenatal care from pregnant women. We included surveys if they were done during or after 2004. No intervention coverage data were available in this period for Botswana, Cape Verde, Comoros, Eritrea, Gabon, South Africa, and south Sudan. We matched all estimates of insecticide-treated nets and intermittent preventive treatment coverage to digitised administrative boundaries reported by the national surveys with sources and methods described previously using ArcGIS 9.1 (ESRI, NY, USA).^18^

Study populations for insecticide-treated net use included pregnant women, women aged 15–49 years, and, for three surveys (Côte d'Ivoire, Malawi, and Mozambique), women who had a pregnancy in the 2 years before the survey.^19^, ^20^, ^21^ Sample sizes in pregnant women at the ADMIN1 level were frequently small. To increase the power of the analysis for coverage, we computed the correlation between insecticide-treated net use in pregnant women and women aged 15–49 years (webappendix pp 1–2). This correlation was strong (*r*^2^=0·9, p<0·0001) and therefore we used coverage among women aged 15–49 years as a proxy for coverage among pregnant women. We defined coverage from survey data as reported use of an insecticide-treated net the night before the survey; most surveys defined an insecticide-treated net as one that was treated in the past 12 months or a longlasting insecticide-treated net. Three surveys (Djibouti, Mauritania, and north Sudan) used insecticide treatment in the previous 6 months to define insecticide-treated nets.^22^, ^23^, ^24^

Study populations for intermittent preventive treatment included women aged 15–49 years who gave birth in the previous 2 or 5 years for the last birth, pregnant women, or women with a birth in the past year. Intermittent preventive treatment was defined as sulfadoxine–pyrimethamine provided at predefined intervals during pregnancy. WHO recommends that all pregnant women in stable malaria transmission areas receive at least two doses of sulfadoxine–pyrimethamine, at the first and second routine antenatal clinic visit after quickening (first baby movements felt by the mother), and at least 1 month apart.^3^ The survey indicator closest to this definition was at least two doses of sulfadoxine–pyrimethamine during an antenatal clinic visit. However, only six countries used this definition;^24^, ^25^, ^26^, ^27^, ^28^, ^29^ 13 other countries used a broader definition of at least two doses of sulfadoxine–pyrimethamine, of which at least one was from the antenatal clinic. We therefore categorised intermittent preventive treatment into four indicators: first, at least one dose from any source; second, at least one dose from an antenatal clinic; third, two or more doses from any source; and fourth, two or more doses, at least one of which was received during an antenatal clinic visit. The first indicator was the most commonly available (39 data sources) and was therefore used to map coverage of intermittent preventive treatment. For Niger and north Sudan, data were only available for the third indicator so these were used as a proxy for the first indicator.

**Figure 1:**
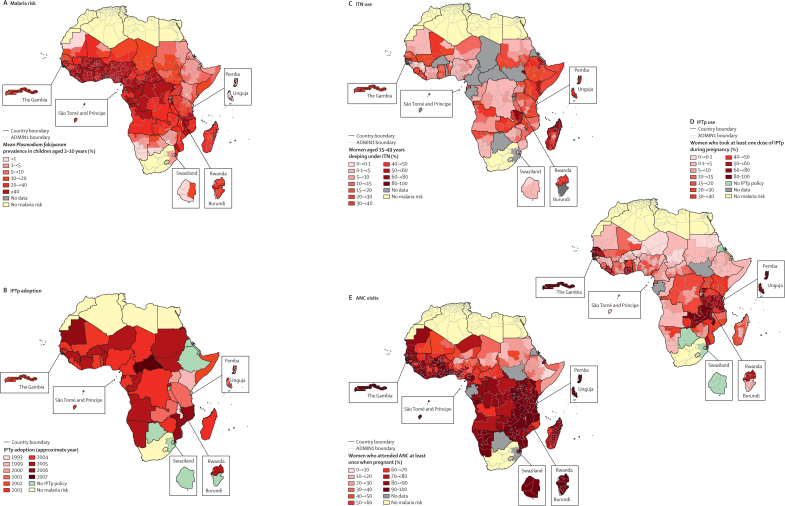
Malaria risk (A), IPTp policy adoption (B), ITN coverage in women aged 15–49 years (C), IPTp coverage of at least one dose of sulfadoxine–pyrimethamine from any source (D), and ANC coverage (E) in countries in sub-Saharan Africa Ethiopia and Burundi have no IPTp policy (D), but data were collected for sulfadoxine–pyrimethamine use in pregnant women in the last-available survey; Mauritania, Congo, and the Central African Republic had no IPTp policy at the time of the survey; Chad and Guinea adopted IPTp <1 year before the survey. ADMIN1=first-level administrative unit. IPTp=intermittent preventive treatment in pregnancy. ITN=insecticide-treated net. ANC=antenatal clinic.

We used published data for the limits and intensity of *Plasmodium falciparum* transmission (webappendix pp 3–4)^30^, ^31^, ^32^, ^33^ that defined malaria risk in 2007 on the basis of a continuous scale of predicted annual mean prevalence in children aged 2–10 years (*Pf*PR_2–10_), and categorised ADMIN1 regions as high-intensity transmission (*Pf*PR_2–10_ ≥40%), medium-intensity transmission (*Pf*PR_2–10_ 10–39%), or low-intensity transmission (*Pf*PR_2–10_ <10%; figure 1A).

To obtain estimates of the number of births per year, we used the national estimates of the annual number of livebirths for 2005–10 from the 2008 revision of the population database of the UN Population Division,^34^ and added estimates of stillbirths projected for 2007 as reported previously.^2^ To obtain population size by ADMIN1 for 2007, we used the population data from the Global Rural Urban Mapping Project, providing 1×1 gridded population counts in ArcView 3.2 (ESRI) as described in detail previously.^31^, ^35^ We distributed the estimated total number of pregnancies at risk of malaria in proportion to the estimated 2007 population at risk of malaria (*Pf*PR_2–10_ >0) by ADMIN1; we excluded an estimated 122 000 pregnancies in eight ADMIN1 regions without malaria (Addis Ababa in Ethiopia, Erongo, Hardap, and Karas in Namibia, Hhohho and Shiselweni in Swaziland, and Bulawayo and Harare in Zimbabwe).

### Modelling procedures and assumptions

**Figure 2:**
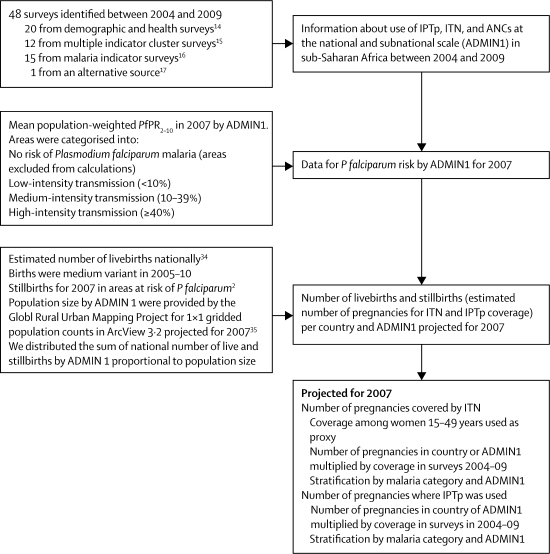
Flow diagram for calculation of number of pregnancies protected against malaria by ITNs or IPTp in sub-Saharan Africa for a hypothetical pregnant population in 2007 IPTp=intermittent preventive treatment in pregnancy. ITN= insecticide-treated net. ANC=antenatal clinic. *Pf*PR_2–10_=predicted annual mean prevalence of Plasmodium *falciparum* in children aged 2–10 years. ADMIN1=first-level administrative unit.

With coverage data obtained from surveys done in 2004–09, we estimated the absolute number of pregnancies that would have been protected or unprotected for malaria in a country at the national scale and by malaria-transmission level for a hypothetical pregnant population in 2007 (figure 2). Because intermittent preventive treatment is provided in the second and third trimester only, the number of pregnancies was defined as the number of livebirths plus stillbirths (ie, induced and spontaneous abortions were not taken into account). Because demographic data for birth rates were not available at all ADMIN1 levels, national statistics were applied to each underlying ADMIN1 level. Each ADMIN1 level was categorised to a single malaria transmission intensity category (eg, differences in malaria categories within an ADMIN1 were not taken into account). Reported insecticide-treated net use in the previous night by women aged 15–49 years was used as an indicator for insecticide-treated net use during pregnancy.

### Statistical analysis

We calculated the median (IQR) coverage of use of insecticide-treated nets, intermittent preventive treatment, and antenatal care by ADMIN1 and stratified these data by the three categories of malaria transmission intensity. We calculated the number of regions with coverage of 60% or more of these interventions and assessed the association between time of policy adoption and high coverage (≥60%).^3^ We compared proportions with the χ^2^ test or the Fisher's exact test if appropriate and p<0·05 was regarded as significant. All data were analysed with SAS version 9.2.

### Role of the funding source

The sponsor of the study had no role in study design, data collection, data analysis, data interpretation, or writing of the report. The corresponding author had full access to all the data in the study and had final responsibility for the decision to submit for publication.

## Results

Apart from Cape Verde and South Africa, all countries had a policy to promote insecticide-treated nets for pregnant women, which had been adopted gradually between 1998 and 2007 (table 1). Most of these countries had adopted such a policy by 2004. For many countries, the dominant mechanisms used to provide insecticide-treated nets to pregnant women were difficult to establish, but all countries stated antenatal clinics as part of their distribution system to target pregnant women.

**Table 1:** Malaria prevention policies for pregnant women in sub-Saharan African countries

	**ANC-Malaria in pregnancy policy**			**ITNs for pregnant women**		**Distribution systems (approximate year adopted)**	**IRS (year adopted)**	**Last survey**
	**Drug (number of doses)**	**Start year**	**Policy ambition**	**Target (year adopted)**	**Policy ambition**			
Angola^10^, ^36^	IPTp with SP (two)	2005	2011: 80% IPT2	Yes (2000)	2012: 80%	FMCN (2005), RFD (ANC 2001), free of charge to vulnerable groups (2001), SPPS (2005)	Yes (2003)	MIS 2006–07
Benin^37^, ^38^	IPTp with SP (two)	2005	2005: 60% (prevention); 2010: 80% IPT	Yes (2002)	2005: 60%; 2010: 80%	FMCN, RFD (ANC), SPPS, HSrp	Yes (2008)	DHS 2006
Botswana^39^	Chloroquine and proguanil	Not clear	2011: 100% in ANC visitors	Yes (2006)	2011: >60%	SPPS, RFD (ANC), HSrp	Yes (1950s)	None available
Burkina Faso^10^, ^40^	IPTp with SP (two)	2005	2010: 80% IPT2	Yes (2004)	2010: 80%	FMCN (2005), RFD (ANC 2005), SPPS (2005), free (2007)	No	MICS 2006
Burundi^41^, ^42^	None	..	..	Yes (2002)	2005: 50%; 2007: 60%; 2010: 80%	FMCN, RFD (ANC), free	Yes (2008)	MICS 2005
Cameroon^10^, ^43^	IPTp with SP (two)	2004	2006: 60% IPT; 2007: 75% IPT; 2014: 80% IPT2	Yes (2003)	2006: 60%; 2014: 80%	FMCN (2007), RFD (ANC 2003), SPPS (2005), free (2003)	No	MICS 2006
Cape Verde^44^	Chloroquine	..	None	No	None	..	No	None available
Central African Republic^45^	IPTp with SP (two)	2007	2005: 60% IPT; 2011: 80% IPT	Yes (2007)	2005: 60%; 2012: 80%	FMCN, RFD (ANC), free	No	MICS 2006
Chad^10^, ^46^	IPTp with SP (two)	2004	2013: 80% IPT in ANC attendees	Yes (2003)	2013: 80%	FMCN (2006), RFD (ANC 2003), free (2003)	No	DHS 2004
Comoros^47^, ^48^	IPTp with SP (two)	2003	2004: 45% IPT; 2014: 80% IPT	Yes (2001)	2005: 50%; 2012: 80%*	FMCN, RFD (ANC 2007), free	Yes (2007)	None available
Congo^49^, ^50^†	IPTp with SP (two)	2006	Not clear	Yes (2004)	Not clear	FMCN, RFD (ANC), free	No	DHS 2005
Côte d'Ivoire 10, 51	IPTp with SP (two)	2005	2011: 80% IPT	Yes (2005)	2011: 80%	FMCN (2006), RFD (ANC 2006), free (2006)	No	MICS 2006
Djibouti^52^, ^53^	None	..	..	Yes (2000)	2010: 80%	FMCN, RFD (ANC), free	Yes (1990s)	MIS 2008–09
DR Congo^10^, ^54^	IPTp with SP (two)	2004	2011: 80% IPT	Yes (2006)	2011: 80%	FMCN (2003), RFD (ANC 2003), free (2006), SPPS (2003)	Yes (2008)	DHS 2007
Equatorial Guinea^55^, ^56^	IPTp with SP (two)	2005	2010: 80% IPT	Yes (2007)	2009: 50%; 2010: 60%	FMCN, RFD, free	Yes (2004)	MIS 2009
Eritrea^57^, ^58^	Chloroquine	2005	2009: 35%‡	Yes (2005)	2007: 80%; 2009: 90%; 2014: 90%	Free to residents of areas with malaria, RFD (ANC)	Yes (2000)	None available
Ethiopia^10^, ^59^	None	..	..	Yes (2001)	2010: 100%	FMCN (2006), SPPS 2004, RFD (ANC 2006), free (2004)	Yes (1997)	MIS 2007; DHS 2005
Gabon^60^, ^61^	IPTp with SP (two)	2003	2007: 60% IPT; 2010: 80% IPT	Yes (2003)	2007: 60%	RFD (ANC 2005), free, FMCN	No	None available
Gambia^62^, ^63^	IPTp with SP (two)	2003	2005: 60% IPT2; 2007: 70% IPT2; 2015: 80% IPT2	Yes (2002)	2005: 60%; 2009: 90%; 2015: 90%*	RFD (ANC 2002), free	Yes (2008)	MIS 2008
Ghana^10^, ^64^, ^65^	IPTp with SP (three)	2003	2010: 60% IPT; 2015: 100% IPT2	Yes (1999)	2010: 60%; 2015: 85%	FMCN (2000), RFD (ANC 1999), SPPS (1997), free (2006)	Yes (2005)	DHS 2008
Guinea^66^, ^67^	IPTp with SP (two)	2005	2006: 80% IPT; 2010: 80% IPT2	Yes (2002)	2006: 30%; 2010: 60%	FMCN, RFD (ANC 2008)	No	DHS 2005
Guinea-Bissau^68^, ^69^	IPTp with SP (two)	2004	2009: 60% IPT; 2011: 80% IPT	Yes (2004)	2009: 60%; 2011: 80%	FMCN, RFD (ANC 2004), free	No	MICS 2006
Kenya^10^, ^70^, ^71^	IPTp with SP (two or three)	1999; 2009	2006: 60% IPT2; 2013: 50% IPT2	Yes (2001)	2006: 60%; 2013: 80%	FMCN (2006), RFD (ANC 2005), SPPS (2002), free (2006)	No	DHS 2008
Liberia^72^	IPTp with SP (two)	2004	2010: 75% IPT; 2013: 95% IPT	Yes (2004)	2010: 60%; 2013: 85%	FMCN, RFD (ANC), free	No	MIS 2008–09
Madagascar^10^, ^73^	IPTp with SP (two)	2004	2010: 80% IPT2; 2012: 80% IPT2	Yes (2000)	2010: 65%; 2012: 80%	FMCN (2007), RFD (ANC 2005), SPPS (2000), free (2004)	Yes (1998)	DHS 2008–09
Malawi^10^, ^74^	IPTp with SP (two)	1993	2010: 90% IPT2	Yes (2002)	2010: 80%	RFD (ANC 2002), free (2006)	No	MICS 2006
Mali^10^, ^75^, ^76^	IPTp with SP (two)	2003	2011: 80% IPT2; 2014: 90% IPT2	Yes (2006)	2011: 80%; 2014: 90%	FMCN (2005), RFD (ANC 2006), SPPS (2005), free (2005)	Yes (2008)	DHS 2006
Mauritania^77^, ^78^	IPTp with SP (two)	2006	2006: 70%; 2010: 80% IPT1	Yes (2002)	2006: 70%; 2010: 80%	RFD (ANC), FMCN, free	No	MICS 2006
Mozambique^10^, ^79^	IPTp with SP (two)	2006	2007: 60% IPT1; 2010: 80% IPT1	Yes (2003)	2007: 90%; 2009: 95%	RFD (ANC 2003), free (2003)	Yes (2005)	MIS 2007
Namibia^80^, ^81^, ^82^, ^83^	IPTp with SP (two); first and second pregnancy	2005	2006: 60% prophylaxis	Yes (2002)	2005: 70%; 2007: 70%; 2010: 70%	FMCN, RFD (ANC), free	Yes (before 2000)	DHS 2006–07; MIS 2009
Niger^10^, ^84^	IPTp with SP (two)	2005	2010: 80% IPT1	Yes (1998)	2010: 80%	FMCN (2005), RFD (ANC 2004), SPPS (2003), free (2005)	No	DHS 2006
Nigeria^10^, ^85^	IPTp with SP (two)	2004	2010: 90% IPT1	Yes (2001)	2010: 80%	FMCN (2006), RFD (ANC 2001), SPPS (2004), free (2001)	No	DHS 2008
Rwanda^27^, ^86^§	IPTp with SP (two)	2005–08	2006: 60% IPT	Yes (2000)	2006: 60%; 2010: 80%; 2012: 85%	FMCN, RFD (ANC), SPPS, free	No	DHS 2007–08
São Tomé and Príncipe 87, 88, 89	IPTp with SP (two)	2004	2010: 90% IPT (G1/G2)	Yes (2004)	Not reported; 2009: 80% of population	FMCN, RFD (ANC), free	Yes (2003)	MICS 2006
Senegal^10^, ^90^	IPTp with SP (two)	2004	2010: 80%	Yes (1998)	2010: 80%	FMCN, RFD (ANC 2005), SPPS 2000, free (1998), HSrp	Yes (2007)	MIS 2008–09
Sierra Leone^91^	IPTp with SP (two)	2004	2008: 60% IPT; 2015: 80% IPT2	Yes (2000)	2008: 40%; 2015: 80%	FMCN (2006), RFD (ANC), free	No	DHS 2008
Somalia^92^, ^93^¶	IPTp with SP (two)	2002	2005: 60% IPT2; 2010: 70% IPT2; 2015: 80% IPT2	Yes (2002)	2005: 60%; 2010: 80%; 2015: 80%	RFD (ANC), free, SPPS, HSrp	No	FSNAU 2008–09; MICS 2006
South Africa^94^	No	..	..	No	..	..	Yes (2000)	None available
Sudan (north)^10^, ^95^	IPTp with SP (two)	2005	2012: 60% IPT	Yes (2001)	2012: 80%	FMCN (2008), RFD (ANC 2007), SPPS (2002), free (2001)	No	MIS 2009
Sudan (south)^10^, ^96^	IPTp with SP (two)	2005	2011: 60% IPT2	Yes (2004)	2011: 60%	FMCN, RFD (ANC)	No	None available
Swaziland^97^, ^98^, ^99^	No‖	..	..	Yes (2002)	2007: 80%	FMCN, RFD (ANC), free	Yes (2000)	DHS 2006–07
Tanzania^10^, ^100^, ^101^	IPTp with SP (two)	2001	2007: 60% IPT2; 2013: 80% IPT2	Yes (2004)	2007: 60%; 2013: 80%	FMCN (2005), RFD (ANC 2004), SPPS (ANC voucher system)	No	MIS 2007–08
Togo^102^, ^103^	IPTp with SP (two)	2003	2006: 70% IPT; 2010: 80% IPT	Yes (2001)	2006: 65%; 2010: 90%	FMCN, RFD (ANC), free	No	MICS 2006
Uganda^10^, ^104^	IPTp with SP (two)	2000	2005: 60% IPT2; 2015: 85% IPT2	Yes (2003)	2005: 60%; 2010: 85%	FMCN (2004), RFD (ANC 2004), SPPS (2004), free (2006), CRps	Yes (2006)	DHS 2006
Zambia^10^, ^105^	IPTp with SP (three)	2001	2008: 80% IPT3; 2011: 90% IPT3	Yes (2000)	2008: 80%; 2011: 80%	FMCN (2003), RFD (ANC 2001), free (2005), SPPS (2001)	Yes (2000)	MIS 2008
Zanzibar^106^, ^107^, ^108^	IPTp with SP (two)	2001	2008: 70% IPT	Yes (2004)	2008: 80%	RFD (ANC)	Yes (2006)	MIS 2007–08
Zimbabwe^109^, ^110^, ^111^	IPTp with SP (two or three)**	2004	2012: 85% IPT2 (in ANC attendees)	Yes (2001)	2004: 50%; 2012: 80%*	FMCN, RFD (ANC), free	Yes (1949)	DHS 2005–06; MIS 2008

Approximate year or ambition information can differ between and within sources. Policy ambition was percentage of coverage aimed at the national scale. IRS in this table is defined as the primary means of vector control, not when used only for prevention and control of epidemics. ANC=antenatal clinic. ITN=insecticide-treated net. IRS=indoor residual spraying. IPTp=intermittent preventive treatment in pregnancy. IPT=intermittent preventive treatment. IPT1=first dose of IPT. IPT2=second dose of IPT. SP=sulfadoxine–pyrimethamine. FMCN=national free mass campaigns. RFD=routine free distribution through public sector. SPPS=subsidised private or public sector. MIS=malaria indicator survey. HSrp=highly subsidised routine distribution through public sector. DHS=demographic and health survey. MICS=multiple indicator cluster survey. G1/G2=Women in their first or second pregnancy. CRps=cost recovery through public sector. FSNAU=Food Security and Nutrition Analysis Unit.

* Either ITN or IRS protected.

† IPTp in Congo needs to have been adopted between 2006 and 2009, ITN for pregnant women needs to have been adopted before 2005, but the year could not be verified from sources. A national strategic plan could not be obtained.

‡ The national malaria control strategy, 2005–10, intended to “provide an opportunity to initiate chemoprophylaxis and IPT for pregnant mothers who live in highly malarious areas only for transmission seasons”;^57^ however, no chemoprevention or IPTp is mentioned in the Global Fund proposal of 2009; no IPTp was implemented.

§ Rwanda stopped IPTp in 2008, because of the changed situation with regards to malaria transmission.^28^

¶ Of the countries in this table, Somalia is the only country that did not sign the Abuja declaration in 2000.

‖ DHS 2006–07 reports intermittent preventive treatment with chloroquine, but this is not confirmed from other sources.^97^

** Whether IPTp with two or three doses is used according to the national malaria policy draft 2008–13 is not clear.^109^

Eight countries (Botswana, Burundi, Cape Verde, Djibouti, Eritrea, Ethiopia, Swaziland, and South Africa) did not have any explicit policies related to the provision of intermittent preventive treatment in pregnancy. Burundi had a policy for screening and treatment during antenatal visits.^41^ Eritrea, which has both *Plasmodium vivax* and *P falciparum* transmission, has kept the option of intermittent preventive treatment in its national policy, but, to our knowledge, this has not been implemented and chloroquine prophylaxis is used.^57^ No drug policies specific to pregnant women were provided in the national guidelines in Djibouti, Ethiopia, Swaziland, or South Africa. All eight countries have a low or unstable risk of *P falciparum* transmission.^31^ The remaining 39 countries had adopted intermittent preventive treatment in pregnancy between 1993 and 2007, mostly between 2004 and 2005 (figure 1B, table 1). Most countries adopted two doses of sulfadoxine–pyrimethamine, although Ghana and Zambia recommended three doses. In 2009, Kenya changed from two to three or more doses as part of focused antenatal care.^112^ Rwanda stopped intermittent preventive treatment in 2008 because of evidence of reductions in malaria transmission.^28^

We obtained data for intervention coverage from 48 surveys done between 2004 and 2009 (12 multiple indicator cluster surveys, 20 demographic and health surveys, 15 malaria indicator surveys, and one survey from the FSNAU in Somalia) in 364 ADMIN1 regions from 40 countries (table 2). 27 (56%) of 48 surveys were done between 2007 and 2009.

**Table 2:** Surveys used and number of women, IPTp coverage, ITN coverage, and ADMIN1 regions in 40 malaria-endemic countries in Africa

	**Source**	**Months**	**% of women attending ANC ≥1 visit (sample size)**	**% of women receiving IPTp ≥1 dose of SP (sample size)**	**ITN use**		ADMIN1 regions*	
					% pregnant worn en (sample size)	% non-pregnant women (sample size)	Number	Number in areas of high-intensity transmission†
Angola‡	MIS 2006–07	Nov–April	79·8% (1422§)	4·7% (1010¶)	22·0% (269)	14·1% (3322)	4	2
Benin	DHS 2006	July–Nov	88·0% (10 521§)	4·9% (6380¶)	19·6% (1962)	18·7% (18 939)	12	10
Burkina Faso‡	MICS 2006	April–June	85·0% (2368¶)	1·8% (2368¶)	..	..	13	13
Burundi‡	MICS 2005‖	Sept–Dec	92·5% (2986¶)	3·0% (2986¶)	..	..	5	0
Cameroon	MICS 2006	May–June	73·7% (2834¶)	8·8% (2834¶)	..	..	10	7
Central African Republic‡	MICS 2006	Oct–Dec	69·3% (4126¶)	12·0% (4085¶)	..	..	16	13
Chad‡	DHS 2004	July–Dec	42·6% (3719§)	0·3% (3719§)	..	..	8	0
Congo	DHS 2005	July–Nov	88·2% (3568§)	2·0% (3568§)	4·2% (666)	6·1% (7137)	3	3
Côte d'lvoire‡	MICS 2006	Aug–Oct	91·0% (3587¶)	11·9% (3587¶)	No data	6·1% (3587**††)	11	11
Djibouti‡	MIS 2008–09	Dec–Feb	80·6% (2104§)	No data	25·2% (163)	13·1% (5829)	6	0
DR Congo‡	DHS 2007	May–Aug	85·3% (5474§)	16·2% (3435¶)	7·1% (1150)	5·3% (10553)	11	6
Equatorial Guinea‡,‡‡	MIS 2009 Bioko MIS 2009 mainland	Aug–Sept May–June	97·3% (339§§) 99·6% (259§§)	29·6% (425§§) 30·0% (320§§)	40·1% (197) 31·3% (227)	35·3% (3290) 32·4% (2282)	6	4
Ethiopia‡	MIS 2007¶¶ DHS 2005‖	Oct–Dec April–Aug	No data 27·6% (7308§)	No data 2·1% (4321¶)	38·7% (568) 1·1% (1181)	37·1% (6657) 1·4% (14 484)	11	0
Gambia‡	MIS 2008‖‖ MICS 2005–06	Aug Dec–March	No data 97·8% (3071¶)	93·7% (979¶) 54·7% (3070¶)	45·0% (402) No data	40·2% (4094) No data	5	0
Ghana‡	DHS 2008	Sept–Nov	95·4% (2099§)	58·2% (1177¶)	19·9% (353)	17·4% (10689)	10	8
Guinea	DHS 2005	Feb–June	82·4% (4447§)	4·5% (4447§)	0·4% (772)	0·2% (8130)	8	3
Guinea Bissau‡	MICS 2006	May–June	77·9% (2506¶)	10·3% (2506¶)	..	..	4	0
Kenya‡	DHS 2008–09	Nov–Feb	91·5% (3973§)	35·5% (2264¶)	49·0% (601)	41·1% (8849)	8	0
Liberia	MIS 2008–09	Dec–March	95·3% (2687§)	57·9% (1573¶)	32·9% (471)	29·0% (4769)	6	6
Madagascar‡	DHS 2008–09	Nov–July	86·3% (8662§)	11·8% (4807¶)	46·2% (1425)	43·3% (17 917)	22	3
Malawi‡	MICS 2006	July–Nov	91·9% (10552¶)	80·7% (10 552¶)	No data	25·6% (10 552¶***)	3	0
Mali‡	DHS 2006	April–Sept	70·4% (9087§)	16·1% (5663¶)	28·9% (1896)	27·8% (15 326)	9	5
Mauritania‡	MICS 2007 DHS 2003–04	May–Sept Aug–Feb	75·4% (3533¶) No data	No data 0·5% (2366§)	No data No data	No data 2·0% (5211)	5	0
Mozambique‡	MIS 2007	June–July	87·9% (3093§)	31·4% (1099§§)	7·3% (589)	8·7% (1971¶***)	11	3
Namibia	MIS 2009††† DHS 2006–07	April–June Oct–March	Not used 94·6% (3898§)	Not used 27·8% (2054¶)	25·9% (194) 8·8% (541)	24·8% (3282) 6·8% (10626)	13	0
Niger	DHS 2006	Jan–July	46·4% (6301§)	0·3% (6301§)	6·7% (1311)	6·1% (8924)	8	0
Nigeria‡	DHS 2008	June–Oct	57·7% (17 635§)	10·9% (11 027¶)	4·8% (3397)	3·9% (33 705)	37	37
Rwanda‡	DHS 2007–08	Dec–April	95·8% (3658§)	53·0% (2267¶)	60·3% (673‡‡‡)	45·2% (7370‡‡‡)	5	0
São Tomé and Príncipe‡	MICS 2006	May–June	97·3% (1231¶)	0·0% (1231¶)	..	..	2	0
Senegal‡	MIS 2008–09	Dec–Jan	93·9%** (5406¶)	78·1% (5406¶)	28·5% (2949)	25·3% (20 425)	10	0
Sierra Leone‡	DHS 2008	April–June	86·9% (4103§)	19·5% (2478¶)	27·2% (614)	26·5% (7925)	4	3
Somalia‡	FSNAU 2008–09 MICS 2006	Jan–Dec Aug–Sept	No data 26·1% (2325¶)	No data 2·2% (2325¶)	No data No data	20·9% (10 601) No data	3	0
Sudan (north)‡	MIS 2009	Oct–Nov	31·3% (1966§§)	2·5% (1966§§)	17·2% (643)	11·6% (7595)	15	0
Swaziland‡	DHS 2006–07	July–Feb	97·1% (2134§)	No data§§§	0·9% (296)	0·3% (5503)	4	0
Tanzania‡	MIS 2007–08	Oct–Feb	97·0% (4995§)	58·4% (2967¶)	26·0% (823)	24·5% (9189)	21	4
Togo‡	MICS 2006	May–June	84·1% (1627¶)	23·2% (1627¶)	..	..	5	5
Uganda‡	DHS 2006	May–Oct	93·5% (5035§)	36·6% (3247¶)	10·0% (1019)	10·1% (9026)	9	2
Zambia	MIS 2008 DHS 2007	April–May April–Oct	No data 93·7% (4136§)	80·0% (2391§) 86·8% (2631¶)	43·2% (416) 32·7% (773)	38·9% (4550) 28·2% (7390)	9	0
Zanzibar	MIS 2007–08	Oct–Feb	95·7% (131§)	78·4% (77¶)	51·3% (23)	43·5% (313)	2	0
Zimbabwe‡	MIS 2008¶¶¶ DHS 2005–06	No data Aug–March	No data 94·2% (4099§)	No data 12·1% (2144¶)	5·6% (no data) 3·2% (584)	No data 3·0% (8863)	10	0

Botswana, Eritrea, Cape Verde, Comoros, south Sudan, and South Africa were not included because of lack of recent reports (Botswana last report MICS 2000; Comoros MICS 2000; Eritrea DHS 2002, South Africa DHS 2003) or lack of a recent report with malaria information (Cape Verde DHS 2005). Eight countries had no recent data for ITN use in women aged 15–49 years or pregnant women. ANC = antenatal clinic. IPTp = intermittent preventive treatment in pregnancy. SP = sulfadoxine–pyrimethamine. ITN = insecticide-treated net. ADMIN1 = first level administrative region. MIS = malaria indicator survey. DHS = demographic and health survey. MICS = multiple indicator cluster survey. .. = not included in questionnaire. FSNAU = Food Security and Nutrition Analysis Unit Somalia (data used from 2008 and 2009).

* Second-level administrative units used in Nigeria, Madagascar, and Tanzania; areas divided by malaria transmission endemicity in Angola.

† Mean *Plasmodium falciparum* prevalence ≥40% in children aged 2–9 years as projected for 2007.

‡ MICS4, DHS, or MIS planned in 2009 or 2010.

§ Aged 15–49 years and gave birth in previous 5 years.

¶ Aged 15–49 years and gave birth in previous 2 years.

‖ Burundi and Ethiopia report SP use but is not national policy.

** Calculated from dataset.

†† Women aged 15–49 years who gave birth in the past 2 years were asked about ITN use during their last pregnancy; this was the only survey that did not ask about ITN use during the previous night.

‡‡ Two reports were combined, ITN use in women aged ≥15 years was used as proxy for use in women aged 15–49 years; for IPTp, only percentages were available by area and, for Bioko, percentages were reported for areas that were smaller than the ADMIN1 region targeted for IPTp, so we combined the percentages with the proportional distribution presented for ANC data for these areas.

§§ Aged 15–49 years with a pregnancy in preceding year or pregnant at the time of the survey for Sudan (north) and Equatorial Guinea.

¶¶ Households were included only if at <2500 m altitude.

‖‖ MIS 2008 was inconsistent about IPTp. Coverage of use of any drugs for prevention of malaria in pregnancy was lower than was that for SP, but no raw data were available to reconcile these differences so data are presented as in the report.

*** Women who gave birth in previous 2 years.

††† ANC and IPTp data not used because of small sample sizes (192 in nine regions, only for pregnant women). ITN use only assessed in malarious regions of Caprivi, Kavango, Kunene, Ohangwena, Omaheke, Omusati, Oshana, Oshikoto, and Otjozondjupa.

‡‡‡ Data only for longlasting ITNs.

§§§ IPTp with chloroquine reported. However, Swaziland does not have an IPTp policy with SP or chloroquine.

¶¶¶ Not Bulawayo or Harare regions; only data for percentage of ITN use in pregnant women by ADMIN1 region were available, so we recalculated for women aged 15–49 years with the equation shown in webappendix pp 1–2. IPTp use was not reported.

No recent data for insecticide-treated net use in women aged 15–49 years or pregnant women were available for eight countries (Burkina Faso, Burundi, Cameroon, Central African Republic, Chad, Guinea Bissau, São Tomé and Princípe, and Togo). Data from 32 countries for insecticide-treated net use showed that no country reached 60% coverage in women aged 15–49 years (table 2). The highest coverage for pregnant and non-pregnant women was reported in Rwanda (45·2% and 60·3%, respectively), Zanzibar (43·5% and 51·3%), and Madagascar (43·3% and 46·2%). Apart from Rwanda, none of the countries was close to achievement of its stated policy ambition (table 1). At a subnational level, ten ADMIN1 regions had insecticide-treated net coverage of 60% or more (one region in Ethiopia, seven in Madagascar, one in Senegal, and one in Zambia; figure 1C). Regions with 5 years or more between the adoption of the policy and the survey were more likely to have coverage of 60% or more than were regions in which the policy was adopted less than 5 years ago (10 [6%] of 174 *vs* 0 of 119, p=0·007). Eight of these ten ADMIN1 regions were in areas with medium-intensity malaria transmission and two were in areas with high-intensity malaria transmission.

We selected areas where information was available about insecticide-treated net coverage (32 countries and 293 ADMIN1 regions) and used a 2007 projected population surface to represent the mid-point of survey data undertaken between 2004 and 2009. In these areas with any intensity of malaria risk, we estimated that there were 27·7 million pregnancies, of which a total of 4·7 million pregnant women would have used an insecticide-treated net (17%; table 3). Estimated coverage was significantly lower in high-intensity transmission areas compared with medium-intensity or low-intensity transmission areas (p<0·0001; table 3). 5·9 million unprotected pregnancies were in Nigeria (21% of unprotected population), 2·8 million in Democratic Republic of the Congo (10%), 2·1 million in Ethiopia (8%), 1·3 million in Uganda (5%), and 1·3 million in Tanzania (5%). Survey data were not available to provide information about insecticide-treated net coverage for around 3·2 million pregnancies in 13 countries that had a policy for pregnant women (table 2).

**Table 3:** Coverage of prevention for malaria in pregnancy and antenatal clinic use in a hypothetical pregnant population in 2007 in sub-Saharan Africa, according to class of *Plasmodium falciparum* risk by use of survey estimates from 2004–09

		Estimated number of pregnancies*	**Estimated number of pregnancies covered (%)**†	**Estimated number of pregnancies not covered (%)**	**Number of ADMIN1 regions**	**Median coverage (IQR)**
ITN use (32 countries)		27 674 626	4 702 319 (17·0%)	22 972 307 (83·0%)	293	16·3% (5·6–29·2)
	PfPR_2–10_ <10%	5 926 993	1 672 108 (28·2%)	4 254 885 (71·8%)	59	17·3% (5·6–29·2)
	PfPR_2–10_ 10–39%	10 136 161	1 983 298 (19·6%)	8 152 863 (80·4%)	124	22·3% (7·8–39·9)
	PfPR_2–10_ ≥40%	11 611 472	1 046 913 (9·0%)	10 564 559 (91·0%)	110	8·2% (3·3–21·6)
IPTp: any number of sulfadoxine–pyrimethamine doses from any source						
	All (31 countries)	25 589 128	6 428 875 (25·1%)	19 160 253 (74·9%)	293	17·8% (5·7–47·9)
	PfPR_2–10_ <10%	2 711 228	876 938 (32·3%)	1 834 290 (67·7%)	40	14·6% (3·0–43·7)
	PfPR_2–10_ 10–39%	10 112 159	3 420 269 (33·8%)	6 691 890 (66·2%)	124	30·9% (7·6–62·9)
	PfPR_2–10_ ≥40%	12 765 741	2 131 667 (16·7%)	10 634 074 (83·3%)	129	13·8% (5·9–25·0)
IPTp: ≥2 sulfadoxine–pyrimethamine doses (≥1 from an antenatal clinic)						
	All (19 countries)	20 018 128	2 732 388 (13·6%)	17 285 740 (86·4%)	205	10·2% (3·6–29·2)
	PfPR_2–10_ <10%	2 610 065	472 447 (18·1%)	2 137 618 (81·9%)	38	8·5% (1·3–19·9)
	PfPR_2–10_ 10–39%	7 384 418	1 361 741 (18·4%)	6 022 677 (81·6%)	91	15·7% (5·8–41·2)
	PfPR_2–10_ ≥40%	10 023 645	898 199 (9·0%)	9 125 446 (91·0%)	76	6·1% (3·2–20·9)
≥1 antenatal clinic visit						
	All (40 countries)	30 358 625	21 754 107 (71·7%)	8 604 518 (28·3%)	356	88·4% (68·1–95·2)
	PfPR_2–10_ <10%	5 926 993	2 902 021 (49·0%)	3 024 972 (51·0%)	59	85·5% (30·3–95·1)
	PfPR_2–10_ 10–39%	11 273 261	8 999 510 (79·8%)	2 273 751 (20·2%)	149	90·0% (71·4–95·7)
	PfPR_2–10_ ≥40%	13 158 371	9 852 576 (74·9%)	3 305 795 (25·1%)	148	86·2% (70·4–94·0)
≥1 antenatal clinic visit in countries with data for sulfadoxine–pyrimethamine						
	All (31 countries)	25 589 128	19 784 707 (77·3%)	5 804 421 (22·7%)	293	90·0% (72·6–95·7)
	PfPR_2–10_ <10%	2 711 228	2 026 713 (74·8%)	684 515 (25·2%)	40	92·8% (31·9–95·7)
	PfPR_2–10_ 10–39%	10 112 159	8 221 657 (81·3%)	1 890 502 (18·7%)	124	91·7% (79·4–96·5)
	PfPR_2–10_ ≥40%	12 765 741	9 536 337 (74·7%)	3 229 404 (25·3%)	129	88·4% (72·5–95·1)

ANC = antenatal clinic. IPTp = intermittent preventive treatment during pregnancy with sulfadoxine–pyrimethamine. ITN = insecticide-treated net. *Pf*PR_2–10_ = mean prevalence of *Plasmodium falciparum* malaria in children aged 2–10 years (projected for 2007).

* Because of an absence of data for number of births by region, we distributed annual estimated births per country proportionally to population size by region.

† χ^2^ test by malaria transmission level: all comparisons were p<0·0001 apart from ≥1 ANC visits in countries with data for sulfadoxine–pyrimethamine, in which there was no significant difference between areas of low versus high transmission (p=0·09).

For the first definition of coverage with intermittent preventive treatment (any number of sulfadoxine–pyrimethamine doses from any source), national data were available for 36 (92%) of 39 countries that had adopted an intermittent preventive treatment policy; no data were available for Comoros, Gabon, and south Sudan. Additionally, data were available for two countries which had not adopted intermittent preventive treatment (Burundi and Ethiopia). Three countries had not yet started intermittent preventive treatment at the time of the survey (Central African Republic, DR Congo, and Mauritania) and two countries had started less than 1 year before the survey (Chad and Guinea). These five countries are shown in figure 1D, but are not included in the evaluation of intermittent preventive treatment.

With this definition of intermittent preventive treatment, five countries had reached national coverage of more than 60%: The Gambia (99%), Malawi (81%), Zambia (80%), Senegal (78%), and Zanzibar (78%; table 2). At a subnational level, the median coverage of intermittent preventive treatment was 17·8% (IQR 5·7–47·9%; 293 regions) in 31 countries with such a policy in place for 1 year or more at the time of the survey. 49 ADMIN1 regions had coverage of 60% or more (figure 1D), including all regions in The Gambia, Malawi, and Zanzibar, six regions in Ghana, three in Liberia, one in Mozambique, nine in Senegal, 12 in Tanzania, and eight in Zambia. Seven (14%) of 49 ADMIN1 regions with 60% or more intermittent preventive treatment coverage were in low-intensity transmission areas, 32 (65%) in areas of medium-intensity transmission, and ten (20%) in high-intensity transmission areas. Such regions were more likely to be in areas of low-intensity or medium-intensity transmission than in areas of high-intensity (39 [24%] of 164 *vs* ten [8%] of 129; χ^2^ test p=0·0005). Regions that had 5 years or more between policy adoption and survey were more likely to have a coverage of 60% or more compared with regions where the policy was adopted more recently (25 [48%] of 52 *vs* 24 [10%] of 241; χ^2^ test p<0·0001). For seven countries (Angola, Benin, Burkina Faso, Côte d'Ivoire, Namibia, Niger, and Zimbabwe), the definition of the study population (women who gave birth in the previous 2–5 years) included time that occurred before the adoption of the policy, meaning progress in intermittent preventive treatment coverage might have been underestimated. Exclusion of these seven countries resulted in a median coverage of 23·9% (IQR 9·8–54·2%) in the other 227 regions from 24 countries.

When we extrapolated these data for pregnancies in areas of any malaria risk in 2007, we estimated that there were 25·6 million pregnancies with reported information about sulfadoxine–pyrimethamine use (table 3), of which 6·4 million (25%) would have been protected by at least one dose of this drug. Estimated coverage was significantly lower in high-intensity transmission areas than it was in areas of medium or low intensity (table 3). Among unprotected pregnancies, 5·4 million (21%) were in Nigeria, 2·4 million (10%) in DR Congo, and 1·1 million (4%) in north Sudan. We were unable to make any predicted coverage estimates for 1·6 million pregnancies occurring in eight countries without survey data but with an intermittent preventive treatment policy.

For the second definition (at least one dose of sulfadoxine–pyrimethamine from an antenatal clinic), data were available for 215 regions in 21 countries. 60% or more coverage was reported in 43 ADMIN1 regions in seven countries; median coverage was 19·0% (IQR 4·9–50·5%).

For the third definition (at least two doses from any source), data were available for 257 regions in 28 countries with a policy for 1 year or more. Median coverage was 12·0% (IQR 5·4–29·1), and 13 regions in five countries had a coverage of 60% or more (one each in The Gambia, Ghana, and Liberia, three in Senegal, and seven in Zambia).

**Figure 3:**
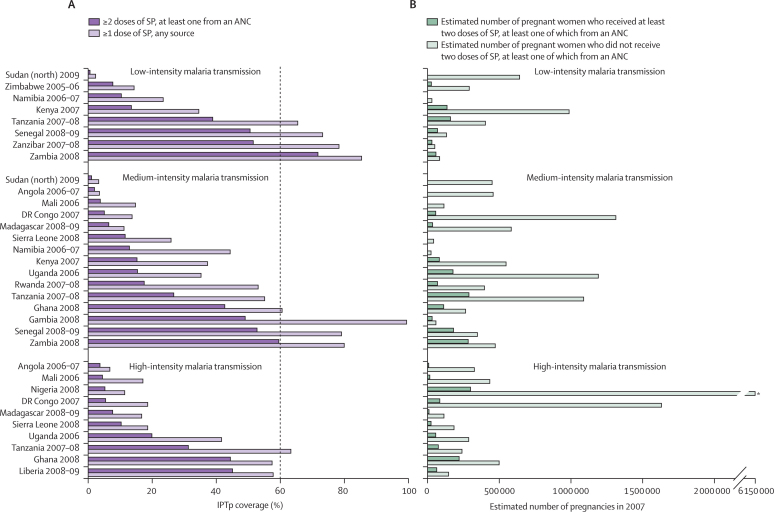
Coverage of SP intermittent preventive treatment (A) and estimated number of pregnant women who received at least two doses of SP, one from an ANC, projected for 2007 (B) SP=sulfadoxine–pyrimethamine. ANC=antenatal clinic. DR Congo=Democratic Republic of the Congo. *Nigeria: 6 150 000 pregnancies per year.

For the fourth definition (at least two doses, one from an antenatal clinic), data were available for treatment coverage for 19 countries and 205 regions; these data are stratified by intensity of malaria transmission in figure 3 (the first definition is provided for comparison). Only Zambia had reached national coverage of 60% or more. 11 ADMIN1 regions had coverage of 60% or more, five regions in Zambia, three in Senegal, and one each in Liberia, Ghana, and The Gambia. Two (18%) of these 11 regions were in high-intensity malaria transmission areas, eight (73%) were in medium-intensity transmission areas, and one (9%) was in a low-intensity transmission area (χ^2^ test p=0·2). There was a strong correlation between intermittent preventive treatment defined as any number of doses from any source and at least two doses of which one from the antenatal clinic (*r*^2^=0·9, p<0·0001; webappendix pp 1–2).

Antenatal clinics are important distribution points for insecticide-treated nets and intermittent preventive treatment. Overall, most pregnant women in sub-Saharan Africa make at least one antenatal clinic visit (figure 1E), with a median of 88·4% (IQR 68·1–95·2; 40 countries, 356 ADMIN1). Six countries had antenatal clinic attendance of less than 60% (table 2), which will be a major bottleneck to achievement of 60% coverage of intermittent preventive treatment; these countries included Chad (42·6%), Ethiopia (27·6%, although there is no intermittent preventive treatment policy), Niger (46·4%), Nigeria (57·7%), Somalia (26·1%), and north Sudan (31·3%). There were substantial inter-regional differences within Nigeria, with antenatal clinic coverage varying from 12·4% in Kebbi State to 97·6% in Anambra State.^113^ All 49 regions with intermittent preventive treatment coverage of 60% or more (≥1 dose, any source) were in areas with high antenatal clinic coverage (≥80%), whereas 154 of 244 regions (63·1%) with intermittent preventive treatment coverage of less than 60% were also in areas with high clinic coverage (p<0·0001).

Projected for 2007, 25·6 million pregnancies occurred in areas of malaria risk in which data were available for antenatal clinic and intermittent preventive treatment (one dose from any source) coverage. Of these women, 19·8 million would have visited an antenatal clinic at least once (77·3%). With the assumption that 6·4 million women received sulfadoxine–pyrimethamine (≥1 dose) from their visit, 13·4 million pregnant women missed an opportunity to receive sulfadoxine–pyrimethamine despite making an antenatal clinic visit (52% of total; 68% of clinic attendees) for the midsurvey period (2004–09), and 5·8 million (23%) women did not visit an antenatal clinic at all. However, this is a conservative estimate, because pregnant women might have received the drugs from other sources than the antenatal clinic.

## Discussion

We find it encouraging that, of 47 sub-Saharan countries assessed, 45 had an insecticide-treated net policy and 39 had an intermittent preventive treatment policy for pregnant women. However, from analysis of surveys, it is sobering to learn that, projected for 2007, in 32 countries with a national policy for an insecticide-treated nets, an estimated 23 million pregnancies were unprotected by an insecticide-treated net, with no information for 3·2 million pregnancies in 13 countries. In 31 countries with an intermittent preventive treatment policy, 19 million were unprotected by intermittent preventive treatment, and there was no information for 1·6 million pregnancies in eight countries with this policy (panel).

Panel **Research in context**

**Systematic review**

We searched PubMed and the Malaria in Pregnancy Library with the terms “insecticide treated nets” or “intermittent preventive treatment”, and “pregnancy” and “Africa”. We identified three studies that reviewed the use of intermittent preventive treatment in pregnancy (IPTp) or insecticide-treated nets (ITNs) in pregnant women at a national scale: one review in 2006 assessing progress of IPTp in five countries with nationwide implementation, containing national survey from two countries (Malawi demographic and health survey 2000, and Kenya demographic and health survey 2003, two or more doses of sulfadoxine–pyrimethamine from any source or during antenatal clinic visit, 29% and 4%, respectively);^11^ one study on the association between ITN use among pregnant women and children, containing data from 15 surveys done in 2003–06 (the proportion of pregnant women who slept under an ITN the previous night ranged from 1·1% to 19·7%);^114^ and one study that reported on equity, and contained data from 22 countries for use of IPTp (two or more doses) or an antimalarial drug during 2006–08 (the range of coverage was not presented, but six countries [Zambia, Senegal, Malawi, Tanzania, Ghana, and The Gambia] exceeded 20% coverage).^115^ Additionally, IPTp and ITN coverage data for pregnant women are often presented in progress reports by WHO, the Roll Back Malaria Partnership, and United Nations Children's Fund. For example, a UNICEF report^116^ presented survey data for 2004–08 for ITN use among pregnant women for 19 countries (coverage 1–60%), and on IPTp use for 24 countries (indicators used were not specified, coverage 0–60%).

**Interpretation**

We provide a synthesis of national survey data for the coverage of malaria prevention in pregnant women in sub-Saharan Africa during 2004–09 in the context of the status of country policies, including a critical appraisal of the survey indicators used. Our study is the first to assess coverage of IPTp and ITNs subnationally, stratified by malaria endemicity, and to estimate coverage at the population level.

In 2000 in Abuja, Nigeria,^3^ African governments pledged to implement measures to ensure that 60% of pregnant women in malaria-endemic areas had access to effective prevention interventions by 2005. By 2006, nationally representative coverage data for the core indicators for intermittent preventive treatment was only available for three countries,^11^ although insecticide-treated net coverage was more commonly reported. 10 years after the Abuja declaration, most malaria-endemic countries in sub-Saharan Africa have adopted intermittent preventive treatment and insecticide-treated nets as interventions for prevention of malaria in pregnancy and the number of countries with nationally representative coverage data for either intervention has increased to 40 of 47 countries. However, very few countries have reached either the targets for 2005 set at the Abuja meeting^3^ in 2000 or their own policy ambition, and countries are even further away from the more recent Roll Back Malaria Initiative targets set for 2010, calling for 80% coverage of insecticide-treated nets in all populations and 100% coverage of intermittent preventive treatment in pregnancy.^9^ Additionally, coverage was lower in areas with high-intensity malaria transmission than it was in areas of middle or low intensity, where women are most in need and conditions for which most empirical trial data support use.

In general, low coverage with intermittent preventive treatment and insecticide-treated nets contrasts with high antenatal-clinic attendance, with a median of 90% by ADMIN1 region for the countries with an intermittent preventive treatment policy for a year or more. This finding suggests that there are missed opportunities when women attended clinics but are not given intermittent preventive treatment (or insecticide-treated nets). Data for frequency of visits or month of start of antenatal-clinic visits were only routinely collected and reported at the national level in the demographic and health survey, so we could not establish the precise number of missed opportunities by assessment of number of women who visited an antenatal clinic at least twice or who started antenatal clinic use before the third trimester. So far, several studies have investigated the factors affecting access and uptake of intermittent preventive treatment. Factors identified included unclear messages about intermittent preventive treatment in pregnancy, especially about timing of the doses, sulfadoxine–pyrimethamine stockouts, limited understanding of intermittent preventive treatment, late enrolment or irregular antenatal clinic visits, and nurse underachievement.^12^, ^117^, ^118^, ^119^, ^120^ Intermittent preventive treatment in pregnancy is not as easy to implement as was initially expected; experiences with it and insecticide-treated net implementation have been described in case reports and used to develop training materials.^12^, ^121^, ^122^ Community-based studies show that, although community sensitisation can be used to increase antenatal-clinic attendance and intermittent preventive treatment uptake,^123^, ^124^ direct community-based distribution of intermittent preventive treatment risks diverting women away from antenatal clinics.^125^ The need to change, due to resistance, from sulfadoxine–pyrimethamine to drug combinations that might need more than 1 day for a treatment course will add to the complexities and challenges of delivery, access, and adherence. Together with the decreasing prevalence of malaria in some regions, these events might cause some countries to reconsider the relevance of their intermittent preventive treatment policy, as occurred in Rwanda. Unfortunately there are no guidelines about when the risk–benefit balance no longer favours intermittent preventive treatment, and these are urgently required; once the system has been stopped, reinstating of intermittent preventive treatment with another drug might be difficult.

The Roll Back Malaria Initiative guidelines^126^, ^127^ published in 2004 and revised in 2009 recommend “proportion of pregnant women who slept under an insecticide-treated net the previous night” as the indicator of insecticide-treated net use. Small sample sizes lead to low precision estimates, and a wide range of possible results. We therefore used the indicator of insecticide-treated net use in women of fertile age as a proxy for insecticide-treated net use in pregnant women. Although not ideal, this approach reduced the frequently mentioned disadvantage that women in their early stage of pregnancy and adolescents might not declare themselves pregnant, leading to a distortion of the indicator among pregnant women.^127^ Conversely, wide-scale insecticide-treated net delivery and promotion efforts directed at pregnant women might result in higher rates of use in pregnant women, such that use of this proxy leads to an underestimate of true coverage. There was, however, a strong correlation between these two indicators (webappendix pp 1–2), which should be expected because pregnancy is a comparatively common occurrence in women in the fertile age range in sub-Saharan Africa.

Our definition of insecticide-treated net coverage was insecticide-treated net use during the previous night as reported by women, which has several limitations. Reported use might not be actual use, or use can be seasonal depending on the perceived nuisance of mosquitoes, and thus the time of survey can affect the rates obtained.^128^ Generally, information about source or the age of the net was not available from these national surveys; old nets need to be replaced, nets might have been discarded after the survey, and as such surveys can overestimate coverage. Conversely, new distribution campaigns after the survey could lead to an underestimate of use. Attainment and maintenance of high coverage of treated nets is challenging because of the complexity of logistics, the changing willingness to use nets, and the lifespan of the net.^129^, ^130^ Immunisation campaigns have been effective at quickly reaching vulnerable populations,^131^ but campaigns alone will not be sufficient to expand and sustain coverage in response to the call for universal coverage.^9^, ^132^, ^133^ Although distribution of insecticide-treated nets to pregnant women through antenatal clinics is an attractive option, acquisition will depend on timing of the first antenatal visit, and might leave women unprotected in the susceptible first trimester.^134^

The first Roll Back Malaria Initiative guidelines^126^ for intermittent preventive treatment in vulnerable populations were vague and recommended the “proportion of women who received intermittent preventive treatment for malaria during their last pregnancy” in “women who delivered a live baby within the last 5 years” as the indicator, with no suggestion of number or source of doses. The revised Roll Back Malaria Initiative guidelines were more specific and recommended assessment of “two or more doses of a recommended antimalarial drug treatment during antenatal clinic visits to prevent malaria during their last pregnancy that led to a livebirth within the last 2 years”.^127^ With both these definitions, recall bias could be an issue. The only guidelines specifically developed by WHO for key indicators for malaria in pregnancy do not list intermittent preventive treatment in pregnancy as an indicator to be measured in household surveys.^135^ A uniform approach to measurement of progress in coverage of intermittent preventive treatment is needed, and although the revised guidelines provide greater clarity on what to measure, this is not yet common practice. However, correlation between the different indicators used in this report was good, and might temporarily help countries to make estimations of coverage of intermittent preventive treatment (webappendix pp 1–2). A larger gap between the two indicators can be expected in areas where the antenatal clinic is not the main source of sulfadoxine–pyrimethamine (eg, distribution by shops or health workers).

Routine surveys focus on measurement of intervention-specific coverage indicators; there is no recognised or recommended combined indicator. The results from trials of combined interventions are not uniform, but use of both insecticide-treated nets and intermittent preventive treatment during pregnancy seems to be of benefit.^136^, ^137^, ^138^, ^139^ Treated nets have the additional benefit of providing protection to the mother before, during, and after pregnancy, and potentially to infants.

Because of the time lag between policy adoption and implementation (sometimes as long as 4 years), use of the implementation year in the timeframe would have been preferable to our use of policy-adoption year, but these data were hard to find from available sources. Although we retrieved many data sources to assess the year of policy adoption for treated nets and intermittent preventive treatment for pregnant women, dates often varied among the sources such that assignment of the year of policy adoption was not obvious. Additionally, information was sometimes inconsistent dependent on the source. Both factors might have affected our analysis. A central, publically available database reporting all national policies would be beneficial to future studies. We used available national surveys for this study; the surveys therefore covered different timeframes, and not all these surveys were recent (only nine surveys were done in 2009), limiting our results. Typically, surveys have a 3–5-year cycle staggered across Africa, although malaria indicator surveys are implemented more frequently. Coverage might have improved in the time between the survey and analysis, and our results might underestimate the true situation. If surveys are most likely to occur in countries that are active in malaria control, we might have assessed the most active countries, and therefore we could have overestimated the situation. Malaria indicator surveys are not always publicly available, and might not have the depth of detail that is needed for this type of analysis (eg, surveys were not done at the administrative level desired, or the sample sizes for pregnant women were too small).

Some countries aim to protect vulnerable populations with insecticide-treated nets and indoor residual spraying. Spraying can potentially affect the coverage of insecticide-treated nets; for example, in an area where spraying is done, people might be less motivated to use treated nets. 24 countries reported the use of spraying as a main technique for reduction of the burden of malaria. Most countries adopted indoor residual spraying recently; and only five countries reported use before 2000. Of the surveys we analysed, 13 reported the percentage of houses sprayed in the 12 months before the survey (>40% in Equatorial Guinea, Mozambique, Zambia, and Zanzibar). Apart from Zanzibar, in which insecticide-treated net coverage was 44%, these countries had insecticide-treated net coverage of less than 40% (Pearson's ρ for correlation 0·4; p=0·2). Therefore, on the basis of a few countries, at the national level there does not seem to be an effect of indoor residual spraying on the use of insecticide-treated nets, though coverage for each intervention was generally low. In the future, when indoor residual spraying might be increasingly used, it will be important that this indicator is standardised and documented at national and subnational levels, and separately reported for vulnerable groups, such as children younger than 5 years or pregnant women.^19^

We have used the malaria transmission map for *P falciparum* to assign malaria endemicity to the administrative regions used in the analysis.^31^ Malaria endemicity can change within short distances and the use of *Pf*PR_2–10_ per ADMIN1 might not accurately represent these changes. Additionally, changes in *Pf*PR_2–10_ will occur because of ongoing malaria control efforts, and these might not be represented in the map; the high coverage of Malaria in Pregnancy prevention measured in medium-intensity transmission areas might be related to a decline in malaria transmission associated with improved control efforts. However, the analysis of coverage by malaria transmission strata at the time of the surveys is sufficiently robust to show high-intensity transmission areas in which malaria prevention is most needed. These maps of malaria risk are often updated and refined to provide more precise spatial and temporal resolutions that will provide a future tracking method to examine inequities in intervention coverage by intensity of *P falciparum* transmission.

Thus, most countries in sub-Saharan Africa have adopted national policies aimed at reduction and control of malaria in pregnancy. The periodicity of routine survey data collection means that up-to-date information is scarce, but from the surveys included in our analysis, with some notable exceptions, we show not enough progress has been made towards the new Roll Back Malaria Initiative goals or the policy ambitions of each country. With only 5 years in which to meet the Millennium Development Goals (and specifically for malaria, goals 4, 5, and 6),^140^ coverage rates of two key interventions are not on course in most countries in sub-Saharan Africa to meet targets. The largest differences in coverage rates could be made in DR Congo and Nigeria.

## Web Extra Material

Supplementary webappendix
