# Supplementary material for: Coverage of malaria protection in pregnant women in sub-Saharan Africa: a synthesis and analysis of national survey data
Source: Lancet Infect Dis. 2011 Mar;11(3):190–207. doi: 10.1016/S1473-3099(10)70295-4 (PMC3119932; doi:10.1016/S1473-3099(10)70295-4)
Supplement: Supplementary webappendix [file mmc1.pdf]

THE LANCET Infectious Diseases

Supplementary webappendix

This webappendix formed part of the original submission and has been peer reviewed. We post it as supplied by the authors.

Supplement to: van Eijk AM, Hill J, Alegana VA, et al. Coverage of malaria protection in pregnant women in sub-Saharan Africa: a synthesis and analysis of national survey data. *Lancet Infect Dis* 2011; published online Jan 26. DOI:10.1016/S1473-3099(10)70295-4.

***Supplement 1*: Correlation between ITN use among women aged 15-49 years and pregnant women, and correlation between IPTp as any dose of SP vs. at least two doses, of which at least one was from the ANC**

***Figure S1*: Scatter plot of use of insecticide treated nets among pregnant women versus women aged 15-49 years in 238 regions (ADMIN1) in 27 countries in sub-Saharan Africa with data available and a sample size of a minimum of 20 in 2004-2009**

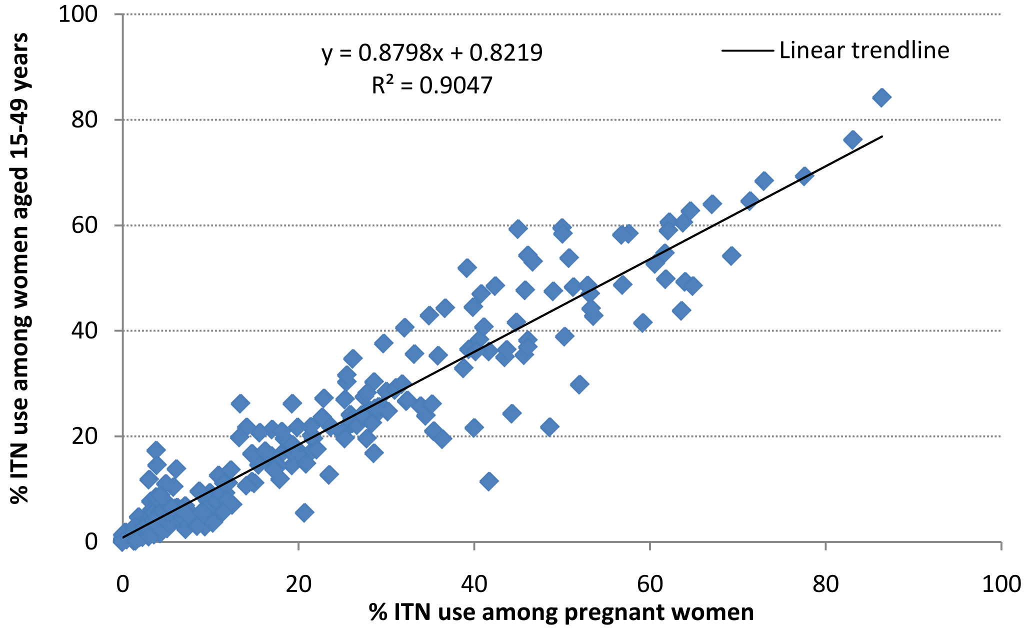

Sample sizes for ITN use among pregnant women by ADMIN1 regions were frequently small; 9% of the 263 ADMIN1 regions with data available had a sample size <20, and 29% had a sample size of 20-49. However, as might be anticipated, the correlation between ITN use among pregnant women and women aged 15-49 years was strong (Figure 1), and since ITN use among women aged 15-49 years was reported more frequently (31 compared to 27 countries) and used larger sample sizes, we used ITN use among women aged 15-49 years as a proxy for ITN use among pregnant women.

Figure S2**: Scatter plot of coverage of IPTp defined as any SP from any source versus at least two doses of SP of which at least one was from the ANC in 220 ADMIN1 regions in 21 countries sub-Saharan Africa with data available, 2004-2009**

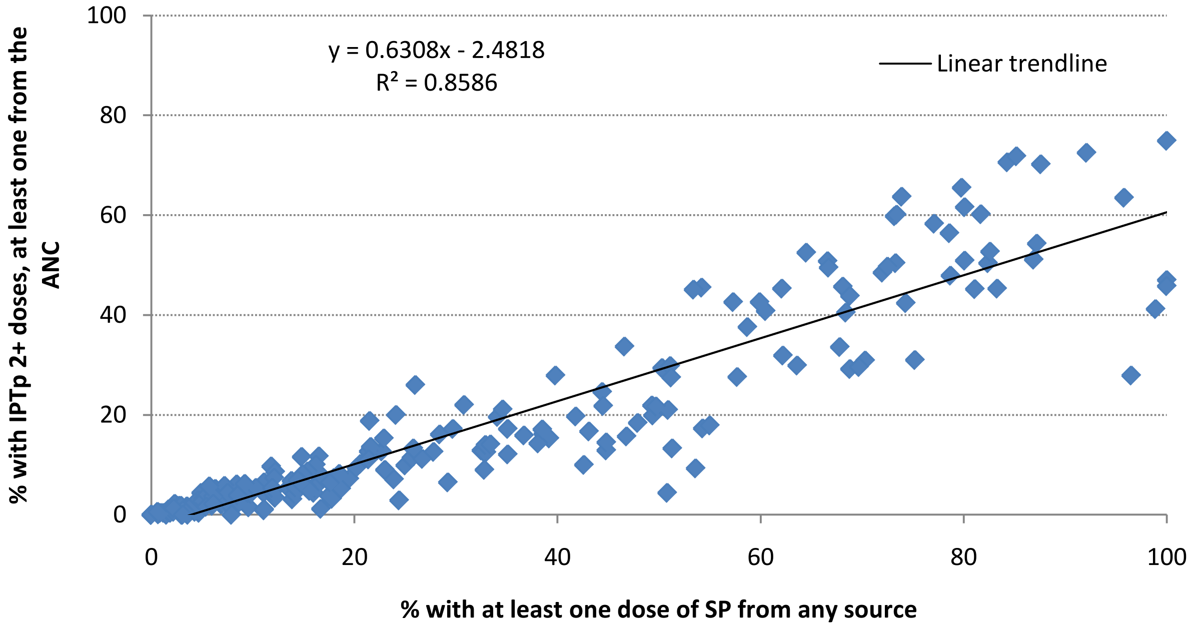

Abbreviations: IPTp: intermittent preventive treatment in pregnancy; SP: sulphadoxine-pyrimethamine

In the 41 surveys with information (Table 2), many indicators were used to assess IPTp coverage, including: percentage of women who took SP (any number of doses), or exactly two doses of SP, or at least two doses of SP, or at least 3 doses of SP from any source; percentage of women who took at least one dose of SP during an ANC visit, or two doses of SP, of which at least one was during an ANC visit, or all during an ANC visit. We categorized these into four definitions: 1) any number of doses of SP from any source; 2) at least 2 doses of SP from any source; 3) any number of doses of SP during an ANC visit; and 4) at least 2 doses of SP received during an ANC visit or of which at least one dose was received during an ANC visit. As can be seen in Figure S2, correlation between IPTp defined as any number of doses from any source and the definition of at least two doses of SP, of which at least one was from the ANC, was good and the definition closest to the description of IPTp. As a rough measure, the indicator "≥2 doses of SP of which at least one was from the ANC" was approximately 0.6 times the most widely used IPTp indicator of "≥1 dose of SP from any source".

Projected for 2007, we calculated an annual number of 20.0 million pregnancies in malaria endemic areas with information on ≥2 doses of IPTp (at least one dose from the ANC) in 19 countries, of which a total of 2.7 million would have used ≥2 doses of SP (13.6%). Estimated coverage would have been significantly lower in the population in high malaria transmission areas (9%) compared to medium or low transmission areas (18%, chi-square test p< , Table 3). No information was available for 7.2 million pregnancies in 20 countries with an IPTp policy.

Supplement 2: Supplementary methods: Geostatistical joint-simulation of ADMIN1-level mean *Pf*PR$_{2-10}$

We use in this study as an areal metric of transmission intensity the predicted 2007 ADMIN1-level population-weighted mean *P. falciparum* parasite rate, age standardised to the two-up-to-ten-years age range. These predictions are derived via two principle methodological steps: (i) the building and fitting of a Bayesian model-based geostatistical (MBG) model that defines an empirical architecture for using a large assembly of parasite-rate observations from community surveys to estimate endemicity at unobserved locations; and (ii) a joint-simulation procedure that allows this model to be implemented over areal units, taking due account of the area-average uncertainty. The methods supporting step (i) are presented in full in Hay *et al.* (1); and those for step (ii) in Gething *et al.* (2), and are outlined in brief here.

Model-based geostatistical modelling of *Pf*PR$_{2-10}$

MBG models take be used to take observations of disease prevalence from dispersed community surveys generate continuous maps by interpolating prevalence at unsampled locations across gridded prediction surfaces (3-5). A principle advantage of MBG in disease mapping applications is its handling of uncertainty. Interpolating sparse, often imperfectly sampled, survey data to predict disease prevalence across wide regions results in inherently uncertain risk maps, with the level of uncertainty varying spatially as a function of the density, quality, and sample size of available survey data, and moderated by the underlying spatial variability of the disease in question. MBG approaches allow these sources of uncertainty to be propagated to the final mapped output, predicting a probability distribution (known formally as a posterior predictive distribution) for the prevalence at each location of interest. Where predictions are made with small uncertainty, these distributions will be tightly concentrated around a central value; where uncertainty is large they will be more dispersed.

Hay *et al.* (1) presented a global map of *P. falciparum* endemicity for 2007, generated from an assembly of 7,953 community parasite surveys collated from 78 countries between 1985 and 2007 used with a Bayesian space-time MBG model to predict urban-adjusted *P. falciparum* parasite rate in the epidemiologically informative 2 up to 10 yr age range, *Pf*PR$_{2-10}$, across a regular spherical grid within the limits of stable transmission (6). The original implementation of this model used an MCMC inference stage to generate 500 samples from the joint posterior distribution of the space-time Gaussian process at the 7,953 locations for which input parasite rate survey data existed $f(x_d, t_d)$, and of a 13 element parameter vector, ϕ. A per-pixel approach was then used to evaluate, for each realisation, values of the Gaussian process at all desired prediction locations $f(x_p, t_p)$, which were then combined with an independently sampled Gaussian random noise component, and subject to an inverse logit transform and multiplication with an age-correction factor to yield the target quantity *Pf*PR$_{2-10}$. The set of realisations of *Pf*PR$_{2-10}$ for each pixel provided an appropriate measure of local uncertainty, with which the precision of *Pf*PR$_{2-10}$ predictions could be assessed at all individual pixel locations worldwide.

Joint-simulation to predict areal averages

Standard approaches to MBG mapping generate suitable metrics of uncertainty for each predicted pixel but do not support the quantification of uncertainty over aggregated regions because between-pixel covariance structures are not retained. Simple aggregation of the per-pixel prediction uncertainties leads to systematically incorrect estimation of the uncertainty associated with aggregated measures, such as the ADMIN1-level means required in this study. Whilst the algebraic solution to this problem in an MBG framework is well known, its large-scale implementation had been prevented by several fundamental computational constraints. Gething *et al.* (2) presented a bespoke approximating algorithm that overcame these problems, allowing the original Bayesian MBG model for *Pf*PR$_{2-10}$ to be implemented globally at 5×5km resolution via joint simulation. in this study, we used this jointly-simulated prediction to generate spatially aggregated mean estimates of *Pf*PR$_{2-10}$ within each ADMIN1-level unit across Africa.

References

(1) Hay SI, Guerra CA, Gething PW, Patil AP, Tatem AJ, Noor AM et al. A world malaria map: *Plasmodium falciparum* endemicity in 2007. PLoS Medicine 2009;6(3):e1000048.

(2) Gething PW, Patil AP, Hay SI. Quantifying aggregated uncertainty in *Plasmodium falciparum* malaria prevalence and populations at risk *via* efficient space-time geostatistical joint simulation. PLoS Comput Biol 2010 April 1;6(4):e1000724.

(3) Diggle P, Ribeiro PJ. Model-based Geostatistics. New York: Springer; 2007.

(4) Diggle P, Moyeed R, Rowlingson B, Thomson MC. Childhood malaria in The Gambia: a case-study in model-based geostatistics. Applied Statistics 2002;51:493-506.

(5) Diggle PJ, Tawn JA, Moyeed RA. Model-based geostatistics. Journal of the Royal Statistical Society Series C-Applied Statistics 1998;47:299-326.

(6) Guerra CA, Gikandi PW, Tatem AJ, Noor AM, Smith DL, Hay SI et al. The limits and intensity of *Plasmodium falciparum* transmission: implications for malaria control and elimination worldwide. PLoS Medicine 2008;5(2):e38.
